# No evidence of sperm conjugate formation in an Australian mouse bearing sperm with three hooks

**DOI:** 10.1002/ece3.577

**Published:** 2013-05-20

**Authors:** Renée C Firman, Blair Bentley, Faye Bowman, Fernando García-Solís Marchant, Jahmila Parthenay, Jessica Sawyer, Tom Stewart, James E O'Shea

**Affiliations:** Centre for Evolutionary Biology, School of Animal Biology, University of Western AustraliaCrawley, Western Australia, 6009, Australia

**Keywords:** Multiple paternity, Muridae, polyandry, sperm competition, sperm morphology

## Abstract

Sperm conjugation occurs when two or more sperm physically unite for motility or transport through the female reproductive tract. In many muroid rodent species, sperm conjugates have been shown to form by a single, conspicuous apical hook located on the sperm head. These sperm “trains” have been reported to be highly variable in size and, despite all the heads pointing in roughly the same direction, exhibit a relatively disordered arrangement. In some species, sperm “trains” have been shown to enhance sperm swimming speed, and thus have been suggested to be advantageous in sperm competition. Here, we assessed the behavior of sperm in the sandy inland mouse (*Pseudomys hermannsburgensis*), a muroid rodent that bears sperm with three apical hooks. First, we accrued genetic evidence of multiple paternity within “wild” litters to unequivocally show that sperm competition does occur in this species. Following this we utilized both *in vitro* and *in vivo* methodologies to determine whether sandy inland mouse sperm conjugate to form motile trains. Our observations of *in vitro* preparations of active sperm revealed that sandy inland mouse sperm exhibit rapid, progressive motility as individual cells only. Similarly, histological sections of the reproductive tracts of mated females revealed no *in vivo* evidence of sperm conjugate formation. We conclude that the unique, three-hooked morphology of the sandy inland mouse sperm does not facilitate the formation of motile conjugates, and discuss our findings in relation to the different hypotheses for the evolution of the muroid rodent hook/s.

## Introduction

Female multiple mating is a taxonomically widespread behavior, and often leads to the ejaculates of different males overlapping in the reproductive tract (Birkhead and Møller 1998; Simmons 2001). In this situation, the sperm of rival males are forced to compete for fertilizations (Parker 1970). A plethora of studies have investigated the implications of female multiple mating, and have provided strong evidence that sperm competition can influence the evolution of male reproductive traits (e.g., Birkhead and Møller 1998; Simmons 2001 and references therein). It is now widely appreciated that selection via sperm competition can generate evolutionary increases in sperm production (e.g., Hosken and Ward 2001; Simmons and García-González 2008; Firman and Simmons 2010) and sperm quality (e.g., Gomendio et al. 2006; Firman and Simmons 2010). However, the role that sperm competition plays in modifying sperm morphologies is still a highly debated topic.

Sperm competition has traditionally been viewed as a race, with the fastest sperm expected to be the most likely to fertilize the ova (Gomendio et al. 2006). Indeed, proficient sperm swimming ability has been shown to be an important predictor of competitive fertilization success in different vertebrate species (Birkhead et al. 1999; Gage et al. 2004; Gasparini et al. 2010; Firman and Simmons 2011), and comparative studies have uncovered positive correlations between sperm length and sperm swimming speed (Fitzpatrick et al. 2009; Lüpold et al. 2009), as well as the level of sperm competition and sperm length (Gomendio and Roldan 1991; Briskie et al. 1997; Byrne et al. 2003; but see Stockley et al. 1997; Immler et al. 2007). More specifically, an analysis on a group of rodents reported a positive association between relative testes size and sperm tail length, and concluded that in some species sperm competition had selected for elongated sperm tails that move faster through the female tract (Breed and Taylor 2000). However, considering most studies of population variation in sperm size have failed to detect a relationship with motility (reviewed Pizzari and Parker 2009), it seems that our understanding of the relationship between sperm size and motility is far from complete, and that sperm competition is more involved than simply a race to the eggs (Roldan et al. 1992; Hunter and Birkhead 2002; Gomendio et al. 2006).

In addition to adjustments in the length of the tail, morphological modifications to other sperm components have been suggested to provide males with a competitive advantage and facilitate the sperms' journey to the site of fertilization (reviewed Higginson and Pitnick 2011). For example, rodents belonging to the Muridae family present a high degree of variation in sperm head morphology; some species exhibit a paddle-shaped ovid form, whilst others bear falciform heads with apical hook/s (Roldan et al. 1992). In species bearing only a single hook it is known that the shape, size, and curvature of the hook vary considerably among species (Breed 2004; Immler et al. 2007), and that hooks often intertwine to form motile sperm trains (Moore et al. 2002; Immler et al. 2007; Fisher and Hoekstra 2010). In European wood mice (*Apodemus sylvaticus*) (Moore et al. 2002), deer mice (*Peromyscus maniculatus*) (Fisher and Hoekstra 2010), and norway rats (*Rattus norvegicus*) (Immler et al. 2007), conjugated sperm trains have been reported to exhibit faster swimming velocities than single cells. Consequently, it was suggested that if sperm conjugates offer a competitive advantage by hastening the advancement toward the ova, then the apical hook might be an evolutionary product of selection via sperm competition (Moore et al. 2002). Indeed, the level of sperm competition was found to predict the length and curvature of the sperm hook among rodent species (Immler et al. 2007). However, intraspecific investigations within the house mouse, *Mus domesticus*, failed to detect the same pattern, and demonstrated that cooperative sperm behavior is not a general adaptation to sperm competition (Firman and Simmons 2009; Firman et al. 2011). Indeed, in the closely related species *Mus musculus*, sperm were shown to form motile conjugates that had reduced swimming velocities compared to individual cells (Immler et al. 2007).

It has also been hypothesized that the muroid sperm hook may facilitate the passage of the sperm to the site of fertilization via enabling their attachment to the epithelia of the female tract (Smith and Yanagimachi 1990). Indeed, sperm-epithelium contact within the oviduct isthmus, which functions as a preovulatory sperm reservoir, has been suggested to be critical for the maintenance of competent sperm fertilizing potential prior to ovulation (Suarez 1987; Smith and Yanagimachi 1990, 1991). A comparative analysis of muroid rodents reported a positive correlation between sperm hookedness and the length of the female receptive period, and thus provided support for the idea that the sperm hook may function to facilitate the attachment of sperm to the oviduct epithelium to allow for the maintenance of their fertilizing potential (Firman and Simmons 2009).

**Figure 1:**
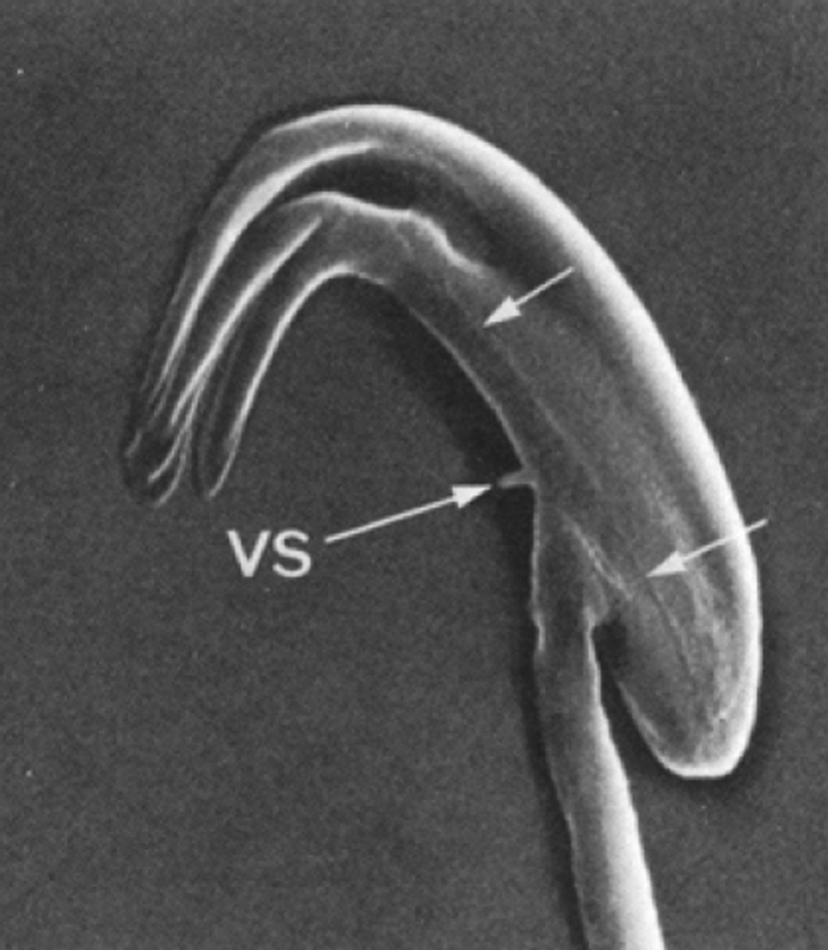
Sperm head morphology of the sandy inland mouse. Scanning electron micrograph from Breed (1983) showing the presence of three hooks, a ridge (arrows), a ventral spike (VS), and a ventral insertion of the connecting piece (×5600).

In this investigation, we explored sperm interactions within the sandy inland mouse (*Pseudomys hermannsburgensis*), a muroid rodent included in the conilurine tribe. Like many of the conilurine rodents, sandy inland mice have falciform sperm heads bearing two ventral and one dorsal hook (Fig. 1, Breed 1983). The sandy inland mouse is a desert-adapted rodent that occurs across most of the Australian continent (Breed and Ford 2007). Currently, very little is known about the reproductive ecology of Australian mice in general. However, like many desert rodents, it is known that the sandy inland mouse inhabits burrows in large, intersexual groups (Breed and Ford 2007). A grouped social organization may facilitate reproduction in the harsh, unpredictable arid environment by ensuring access to mates and/or generating synchronized breeding. Although communal living predisposes individuals to a polygamous mating system, it has not before been documented whether female sandy inland mice mate multiply. Here, we accrued genetic evidence from “wild” litters to show that sperm competition does occur in this species. Following this, we assessed the *in vitro* and *in vivo* behavior of sperm, and looked for evidence of motile train formation. We discuss our findings in relation to the different hypotheses for the evolution of the muroid rodent sperm hook/s.

## Materials and Methods

### Source population and experimental animals

Sandy inland mice were sourced from a wild population located approximately 25 km south of Laverton, Western Australia (28°37′S, 122°24′E). Male (30) and female (30) mice were trapped in Elliot small mammal traps baited with peanut butter and rolled oats, and transported to the University of Western Australia. Three wild-caught females were observed to be pregnant, and were allowed to give birth in captivity. The animals were held in a constant temperature room (26°C) on a reversed light–dark cycle, housed in large mouse boxes (28 × 74 × 41 cm), and provided with native Australian rodent pellets and water *ad libitum*. To supplement their diet the animals received ∼5 g of large parrot seed three times a week. The wild-caught animals were used to establish a small, captive-bred colony. Male–female pairs were housed together until the female was noted as being pregnant, or for a maximum of 3 weeks. Pregnant females were separated from the males and provided with shredded paper for nesting. Litters were weaned from their mother at 28 days old. The mice were outbred for three generations before being used in this experiment.

### Genotyping and paternity analysis

We looked for evidence of multiple paternity in the litters of wild-caught, pregnant females to unequivocally establish that sperm competition does occur in this species. Litter sizes of sandy inland mice typically range from one to seven, with an average of four (Breed 1990). Here, the females gave birth to four pups (2) and three pups (1). DNA was extracted from 1 mm^2^ of ear tissue of females and their offspring (4 weeks old) using the EDNA HISPEX extraction kit (Fisher Biotec, Subiaco, Western Australia, Australia). Microsatellite markers developed for the western pebble-mound mouse (*Pseudomys chapmani*) had previously been shown to cross-amplify in the sandy inland mouse (Moro and Spencer 2003). We selected five of these microsatellite markers (pPc1A7, pPc9A8, pPc6E8, pPc10E12, and pPc2G6) and obtained labeled forward primers from Geneworks (Hindmarsh, South Australia, Australia) (FAM) and Life Technologies (Foster City, CA) (PET, VIC) and unlabeled primers from Geneworks. For each sample, three polymerase chain reactions (PCRs) were performed in combinations of pPc1A7 + pPc10E12, pPc9A8 + pPc6E8, and pPc2G6. PCRs were performed in 10 μL reactions containing 10 mmol/L Tris (Life Technologies), 50 mmol/L KCL (Life Technologies), 1.5 mmol/L MgCl_2_ (Life Technologies), 200 mmol/L of each dNTP (Life Technologies), 250 nmol/L of each labeled forward primer, 250 nmol/L of each unlabeled reverse primer, 0.5 units of Platinum Taq polymerase (Life Technologies), and ∼100 ng of template DNA. The thermocycling profile for each PCR was 5 min denature at 94°C, 35 cycles of 90°C for 30 sec, 62°C for 20 sec, and 72°C for 45 sec, followed by 72°C for 10 min (Moro and Spencer 2003). PCR products (1.5 μL) were run on a ABI3730 Sequencer, sized using Genescan-500 LIZ size standard, and genotyped using Genemapper software (v3.0; Life Technologies).

The number of sires per litter was estimated by allele counting, which involved identifying and subtracting the maternal allele from each offspring and inferring a paternal allele (Birdsall and Nash 1973). The greatest number of nonmaternal alleles at a single locus was taken as the number of paternal alleles. Multiple paternity is evident if the minimal number of paternal alleles at a locus required to explain the amount of genetic variation in a litter is greater than two (Birdsall and Nash 1973; Baker et al. 1999).

### *In vitro* assays for assessment of sperm train formation

We performed *in vitro* assays to assess the behavior of active, free-swimming sperm in sandy inland mice (6). The protocol for *in vitro* sperm activation in mice has been described previously (Firman et al. 2013). Briefly, the caudal region of the epididymis was removed and repeatedly cut in a 500 μL drop of human tubal fluid that was immersed in mineral oil (Firman et al. 2013). After an initial 10-minute incubation (37°C) period, which allowed the sperm to swim into the medium, the epididymal tissue was removed. The sperm suspension was then incubated for a further 50 min. Sperm concentration and sperm motility parameters were quantified using a CEROS computer-assisted sperm analysis system (version 10, Hamilton and Thorne Research) (Firman et al. 2013). Mean (±SE) values of the sperm traits of males used in the *in vitro* sperm observations are presented in the appendix (Table A1). We prepared a 20 μL aliquot of each sperm suspension to assess sperm behavior. We observed each sperm suspension for 5 min (200× and 400× magnification) using an Olympus BX50 light microscope, and recorded short video clips using an Olympus DP72 camera attached to the microscope (Olympus Australia, Mt Waverley, Vic., Australia).

### Histology for assessment of *in vivo* sperm train formation

At 3 months of age, males and females were paired and used for the experimental matings. The matings were conducted during the dark phase of the light cycle, under a red light. The females were placed in the males' box and then checked for a copulatory plug every 2 h. In many rodent species, the copulatory plug is evidence of a complete ejaculatory series, and therefore a successful mating (Rugh 1968; Breed and Adams 1992). Once a plug was detected the females (5) were sacrificed at different time intervals after mating: (i) >2 h (*n* = 2), (ii) 4–6 h (*n* = 1), (iii) 6–8 h (*n* = 1), and (iv) 8–16 h (*n* = 1). The female reproductive tracts were removed and immediately fixed in 10% buffered formalin. The entire tract was then embedded in paraffin wax and longitudinally serial sectioned (10 μm). The tissue was stained with Gill's Hematoxylin and Eosin, and permanently mounted on slides.

We performed a detailed assessment of sperm within the female reproductive tract by viewing each slide under an Olympus BX50 light microscope (4× and 40× objective). We located the sperm within the tract, and then looked for evidence that the sperm were attached together via the hooks to form trains. We used schematic drawings and digital images of sperm trains observed in other species as references for what we might expect to observe. Thus, whilst scanning the slides we looked specifically for (i) a concentration of overlapping sperm heads (Immler et al. 2007; Fisher and Hoekstra 2010) and (ii) the alignment of a number of sperm heads in the same direction (Immler et al. 2007; Fisher and Hoekstra 2010; Moore et al. 2002). We also looked for evidence that sperm were attached to the epithelium. We took images of *in vivo* sperm using an Olympus DP72 camera attached to the microscope (4× and 20× objective).

## Results

Our paternity analysis revealed that female sandy inland mice do mate polyandrously, and thus males are forced to engage in sperm competition. Allele counting revealed that mixed paternity was evident in two of the three “wild” litters. We observed a maximum of three paternal alleles at each informative loci (pPc1A7, pPc9A8, pPc6E8), indicating that there were at least two sires per litter. The genotypes of females and their offspring are provided online in the appendix (Table A2).

**Figure 2:**
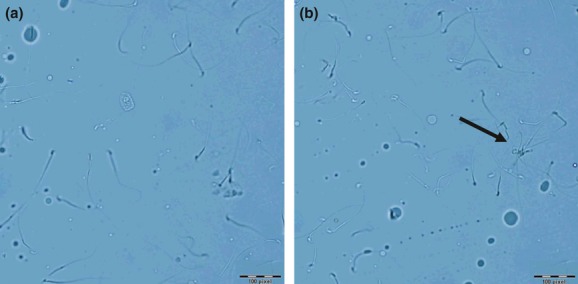
Captured images of active sandy inland mouse sperm. Individual, free-swimming sperm were most often observed (a), however, occasionally nonmotile sperm conjugates were observed (indicated by the arrow; b).

Observations of the in vitro sperm samples revealed no evidence of sperm conjugate formation. We did not observe any active, motile sperm conjugations that could be described as trains. On the contrary, individual, free-swimming sperm were found to exhibit highly progressive, rapid motility (Fig. 2a; example sperm clips are available online in the Supporting Information). Sperm that were clumped together were nonmotile (Fig. 2b).

**Figure 3:**
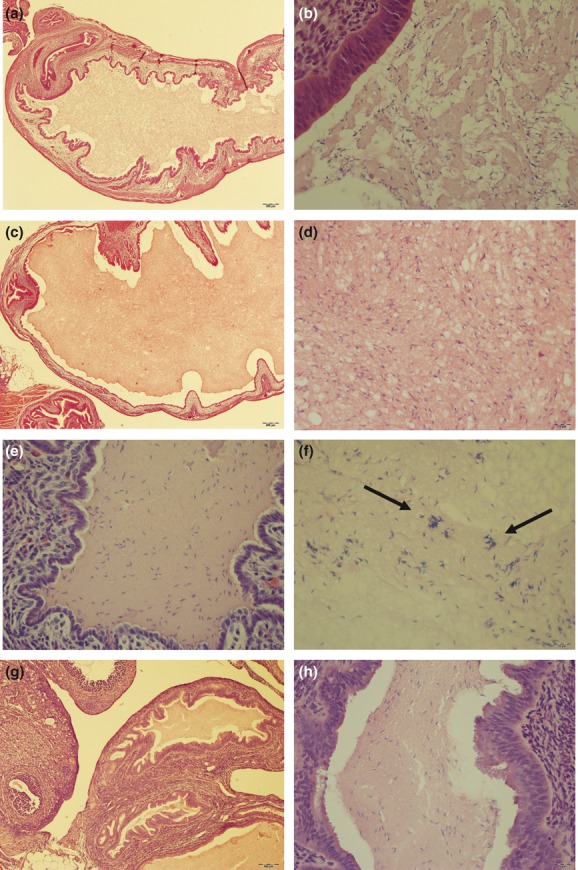
Images of histological sections of the reproductive tract of female sandy inland mice that display *in vivo* sperm interactions. Sperm located in the uterus >2 h after mating at low (a, c) and high (b, d) magnification; sperm located in the lumen and epithelial folds of the uterus 4–6 h after mating (high magnification; e); sperm located in the proximal end of the uterus 6–8 h after mating (high magnification; f); sperm located in the oviduct 8–16 h after mating at low (g) and high (h) magnification. The arrows indicate conjugated sperm.

We found little evidence of sperm train formation in the *in vivo* histological preparations. Within 2 h of mating, individual sperm were seen to be highly concentrated and haphazardly scattered throughout the uteri (Fig. 3a and c). There was no evidence that sperm were conjugated to form trains (Fig. 3b and d). Sperm were found in the convoluted folds of the uterus, but there was no apparent association between the sperm hook and the epithelium (Fig. 3e). Some sperm appeared to be clumped together in small aggregations of 8–20 cells in the upper region of the uterus (Fig. 3f). Only individual sperm were observed in the oviduct (Fig. 3g and h).

## Discussion

Our paternity analysis provided evidence that in nature female sandy inland mice do mate with multiple males, and therefore sperm competition does occur in this species. As our sample size was only three litters, we cannot comment on the frequency or intensity of sperm competition in wild sandy inland mouse populations. We only detected a maximum of two sires per litter. However, given that the size of the litters were small, we may assume that this is a conservative estimate of the number of males that a female will actually mate with in a single reproductive episode. Certainly, receptive female sandy inland mice maintained in the laboratory will readily and actively initiate copulations with more than two males (R. C. Firman, pers. obs.). While the precise duration of estrus in the sandy inland mouse is currently unknown, females of related *Pseudomys* species have been observed to mate over consecutive nights and predicted to be receptive for up to 3 days (Smith et al. 1972). The length of the estrous cycle in the sandy inland mouse may be comparable to other *Pseudomys* species (Watts 1979). Therefore, it is not unreasonable to assume that female sandy inland mice, which live in large, communal groups, have adequate opportunity to mate with many males during their receptive period. Consequently, we can conclude that sperm competition is a prominent selective force in this species.

It has been suggested that sperm train formation may be adaptive under competitive conditions by increasing a male's chance that their sperm will reach the ova first and achieve more fertilizations than their rivals (Moore et al. 2002; Immler et al. 2007; Fisher and Hoekstra 2010; reviewed Higginson and Pitnick 2011). Consequently, we might expect sperm conjugates to form immediately following ejaculation. Indeed, single-hooked sperm of muroid rodent species have been observed to form *in vitro* motile trains within minutes of release from the caudal epididymis (Moore et al. 2002; Fisher and Hoekstra 2010). For example, in the deer mouse *in vitro* trains consisting of ≤40 cells were observed within 1 min of activity, and continued to form for *ca*. 1 h (Fisher and Hoekstra 2010). In contrast, however, we found no *in vitro* evidence of train formation among the three-hooked sperm of the sandy inland mouse. Our analysis of *in vitro* preparations of epididymal sperm revealed that sandy inland mouse sperm exhibit rapid and progressive motility as individual cells only. It could be that variation in *in vitro* sperm train formation across species is attributable to differences in sperm retrieval methodology. However, similar methodologies have been employed across studies; in all cases in vitro sperm behavior was observed in sperm that was harvested from the caudal epididymis and activated in an artificial medium (Moore et al. 2002; Immler et al. 2007; Fisher and Hoekstra 2010). Indeed, within species sperm train formation behavior has been observed to be consistent for both ejaculated and artificially activated sperm (Moore et al. 2002; Firman and Simmons 2009; Fisher and Hoekstra 2010; Firman et al. 2011).

Importantly, we looked at the arrangement of ejaculated sperm *in situ* in the female reproductive tract. Similar to our *in vitro* observations, our histological preparations of *in vivo* sperm returned no evidence of sperm conjugation formation. Sperm within the uterus of females sacrificed within 2 h of mating were seen to be scattered throughout the lumen as single cells only. This observation is in stark contrast to that of the single-hooked sperm of the wood mouse for which the majority of uterine sperm form motile trains and <5% are individual cells (0.5–2 h postmating) (Moore et al. 2002). We did find evidence of conjugated sperm in the proximal end of the uterus of the female sacrificed 6–8 h after mating. However, we suggest that conjugates observed so long after mating are unlikely to be the result of active formation, and more likely to be an artifact of aged sperm clumping together as their progressive motility declines. Certainly, it does not appear that the sperm heads are aligned in the same direction, as would be expected if these conjugates represented motile trains. Consequently, our *in vitro* and *in vivo* preparations of sandy inland mouse sperm have provided no evidence that the sperm hooks function to form conjugated trains. A comparative study provided evidence to suggest that sperm competition may have played a role in the evolution of sperm hooks among the Muridae (Immler et al. 2007). Thus, the absence of sperm conjugate formation in the sandy inland mouse suggests that sperm competition has not driven the evolution of the apical hooks for conjugate formation, or that conjugate formation has been secondarily lost in this species.

In many mammals, sperm reach the site of fertilization long before the ova are released, and are required to retain their motility and fertilizing capacity for many hours prior to fertilization (Gomendio and Roldan 1993). Sperm-oviduct binding, which is thought to be mediated though lectins, has been reported to assist the sperm in maintaining both motility and membrane integrity (Lefebvre et al. 1995; Thomas et al. 1995). Additionally, the influx of calcium, which is a necessary prerequisite for the acrosome reaction, is delayed while sperm are attached to the oviductal epithelial cells (Dobrinski et al. 1996). Effective sperm-oviduct attachment is expected to be under strong selection. *In situ* observations of house mouse sperm in the oviduct revealed that sperm adhere to the epithelium of the lower isthmus via the hook (Suarez 1987). Additionally, among a group of muroid species, a positive correlation was observed between sperm hookedness and the length of the female receptive period (Firman and Simmons 2009). Consequently, whilst the apical sperm hook may function to form sperm conjugates in some species, in others the hook/s may facilitate the attachment of sperm to the oviduct prior to ovulation (Smith and Yanagimachi 1990). In our histological preparations, it appears that sperm have retracted from the oviduct epithelium during the preservation process, so we are unable to comment on the role that the sandy inland mouse sperm hooks might play in facilitating sperm attachment in the oviduct. While observations of sperm located in the proximal end of the uterus revealed that sperm are not necessarily in close association with the epithelium of the female tract, we suggest that additional research is required to properly explore the behavior of three-hooked sperm in the oviduct, and to determine whether the hooks aid sperm attachment to the epithelium.

## Supporting Information

**Table A1**. Mean (±SE) sperm traits values for sandy inland mice used in the *in vitro* sperm observations.

**Table A2**. Genotypes of three female sandy inland mice and their offspring at five microsatellite loci.

**Clips S1 and S2.** Active and motile sandy inland mouse sperm.
