# Supplementary material for: No evidence of sperm conjugate formation in an Australian mouse bearing sperm with three hooks
Source: Ecol Evol. 2013 May 20;3(7):1856–63. doi: 10.1002/ece3.577 (PMC3728929; doi:10.1002/ece3.577)
Supplement: Supplementary file 3 [file ece30003-1856-SD3.docx]

**Appendix**

**Table A1** Mean (± s.e.) sperm traits values for sandy inland mice used in the *in vitro* sperm observations.

| **Trait** | **Mean (± s.e.)** |
| --- | --- |
|  |  |
| Sperm number (x10^6^) | 12.11 ± 1.36 |
| % motile | 78.2 ± 6.2 |
| % progressive | 37.5 ± 4.2 |
| % rapid | 55.7 ± 6.7 |
| Average path velocity (µm/sec) | 103.5 ± 7.5 |

| Straight line velocity (µm/sec) | 79.9 ± 6.0 |
| --- | --- |
| Curvilinear velocity (µm/sec) | 179.6 ± 11.3 |

|  |  |
| --- | --- |

**Table A2** Genotypes of three female sandy inland mice and their offspring at five microsatellite loci.

|  | pPc2G6 | | pPc1A7 | | pPc9A8 | | pPc10E12 | | pPc6E8 | |
| --- | --- | --- | --- | --- | --- | --- | --- | --- | --- | --- |
| L1 | 150 | 159 | 151 | 158 | 217 | 229 | 84 | 88 | 142 | 144 |
| L1_a | 150 | 159 | 151 | 164 | 202 | 229 | 84 | 88 | 142 | 142 |
| L1_b | 150 | 150 | 158 | 158 | 212 | 229 | 88 | 93 | 137 | 142 |
| L1_c | 150 | 150 | 158 | 158 | 212 | 229 | 88 | 93 | 137 | 144 |
| L1_d | 150 | 159 | 158 | 164 | 217 | 229 | 84 | 107 | 142 | 144 |
|  |  |  |  |  |  |  |  |  |  |  |
| L2 | 141 | 148 | 151 | 154 | 210 | 226 | 88 | 93 | 139 | 142 |
| L2_a | 148 | 152 | 147 | 151 | 210 | 217 | 88 | 93 | 132 | 142 |
| L2_b | 148 | 148 | 154 | 154 | 204 | 210 | 93 | 109 | 136 | 139 |
| L2_c | 148 | 152 | 151 | 154 | 222 | 226 | 88 | 109 | 142 | 142 |
| L2_d | 141 | 152 | 151 | 151 | 210 | 217 | 88 | 88 | 132 | 142 |
|  |  |  |  |  |  |  |  |  |  |  |
| L3 | 138 | 148 | 154 | 154 | 210 | 212 | 88 | 93 | 139 | 142 |
| L3_a | 141 | 148 | 154 | 154 | 208 | 210 | 93 | 97 | 139 | 139 |
| L3_b | 134 | 138 | 151 | 154 | 212 | 226 | 93 | 101 | 139 | 142 |
| L3_c | 134 | 148 | 151 | 154 | 208 | 212 | 88 | 97 | 139 | 139 |
